# Is It Possible to Improve Scabies Diagnosis Performance?

**DOI:** 10.5826/dpc.1102a15

**Published:** 2021-04-12

**Authors:** Vincenzo Greco, Massimiliano Scalvenzi, Gabriella Fabbrocini, Matteo Megna

**Affiliations:** 1 Dermatology Unit, Department of Clinical Medicine and Surgery, University of Naples Federico II, Italy

**Keywords:** scabies, mite, feces, eggs, jetliner, jet trail, polarized light, dermoscopy

## Case Presentation

**Figure 1:**
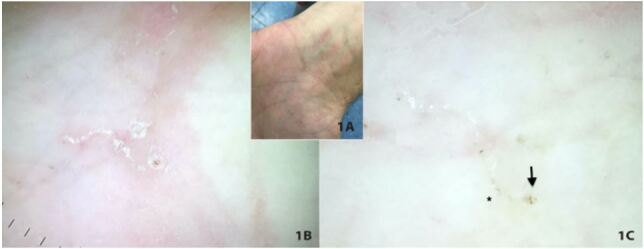
(A) Scabies: clinical aspects. (B) Polarized light dermoscopy. (C) Immersion contact dermoscopy.

We present the same case of scabies (Figure 1A) photographed with a new polarized light dermoscope (Figure 1B) and with an older immersion contact dermoscope (Figure 1C).

## Teaching Point

Dermoscopy allows a high sensitivity and specificity for scabies diagnosis [1]. Although modern dermoscopes with polarized light can better analyze pigmented lesions without necessitating a liquid interface or direct skin contact with the instrument, they do not perform as well when interpreting superficial epidermal lesions of scabies. Note that, with new polarized light dermoscopes, the “jet trail” (the burrow) is easily visible, but a clear differentiation of artefacts induced by scratching or small dirt particles is not easy to obtain [2]. Paradoxically, scabies was more easily detected in the past with older dermoscopes because immersion contact dermoscopy reduces the reflection capacity of keratinocytes of the burrow so that the “jetliner,” feces, and eggs that are usually covered by the shiny keratinocytes of the jet trail, are better detected. In conclusion, we suggest using older dermoscopes or the immersion technique for mite search.

## References

[1] Marghoob AA, Swindle LD, Moricz CZ (2003). Instruments and new technologies for the in vivo diagnosis of melanoma. J Am Acad Dermatol.

[2] Micali G, Lacarrubba F, Verzì AE, Chosidow O, Schwartz RA (2016). Scabies: advances in noninvasive diagnosis. PLoS Negl Trop Dis.

